# Cas3 is a limiting factor for CRISPR-Cas immunity in *Escherichia coli* cells lacking H-NS

**DOI:** 10.1186/s12866-016-0643-5

**Published:** 2016-03-08

**Authors:** Kristina Majsec, Edward L. Bolt, Ivana Ivančić-Baće

**Affiliations:** Division of Molecular Biology, Faculty of Science, University of Zagreb, Horvatovac 102a, 10000 Zagreb, Croatia; School of Life Sciences, University of Nottingham, Medical School, Nottingham, NG7 2UH UK

**Keywords:** CRISPR-Cas, H-NS, PAM, HtpG, Temperature, E. coli

## Abstract

**Background:**

CRISPR-Cas systems provide adaptive immunity to mobile genetic elements in prokaryotes. In many bacteria, including *E. coli*, a specialized ribonucleoprotein complex called Cascade enacts immunity by“ an interference reaction" between CRISPR encoded RNA (crRNA) and invader DNA sequences called “protospacers”. Cascade recognizes invader DNA via short “protospacer adjacent motif” (PAM) sequences and crRNA-DNA complementarity. This triggers degradation of invader DNA by Cas3 protein and in some circumstances stimulates capture of new invader DNA protospacers for incorporation into CRISPR as “spacers” by Cas1 and Cas2 proteins, thus enhancing immunity. Co-expression of Cascade, Cas3 and crRNA is effective at giving *E. coli* cells resistance to phage lysis, if a transcriptional repressor of Cascade and CRISPR, H-NS, is inactivated (Δ*hns*). We present further genetic analyses of the regulation of CRISPR-Cas mediated phage resistance in Δ*hns E. coli* cells.

**Results:**

We observed that *E. coli* Type I-E CRISPR-Cas mediated resistance to phage λ was strongly temperature dependent, when repeating previously published experimental procedures. Further genetic analyses highlighted the importance of culture conditions for controlling the extent of CRISPR immunity in *E. coli*. These data identified that expression levels of *cas3* is an important limiting factor for successful resistance to phage. Significantly, we describe the new identification that *cas3* is also under transcriptional control by H-NS but that this is exerted only in stationary phase cells.

**Conclusions:**

Regulation of *cas3* is responsive to phase of growth, and to growth temperature in *E. coli*, impacting on the efficacy of CRISPR-Cas immunity in these experimental systems.

**Electronic supplementary material:**

The online version of this article (doi:10.1186/s12866-016-0643-5) contains supplementary material, which is available to authorized users.

## Background

*Escherichia coli* K-12 utilises Type I-E CRISPR (Clustered Regularly Interspaced Short Palindromic Repeats) loci to gain immunity to invasive DNA such as bacteriophage (“phage”), dependent on activities of Cas (CRISPR-associated) proteins reviewed in [1–5]. CRISPR loci are composed of the AT-rich leader region followed by arrays of sequence repeats separated by spacers that are homologous to sequences of invading DNA (“protospacers”). CRISPR arrays are transcribed into “pre-crRNA” that is further processed into “crRNA” that contains a full or partial spacer sequence reviewed in [1–5]. In *E. coli*, crRNA assembled into “Cascade” (CRISPR-associated complex for antiviral defence) is targeted to protospacers in “interference” reactions. *E. coli* Cascade comprises five proteins: Cse1 (CasA), Cse2 (CasB), Cas7 (CasC), Cas5 (CasD) and Cas6e (CasE) [6–10]. Interference generates base pairing between crRNA and protospacer DNA in an R-loop, displacing the DNA strand that is not complementary to crRNA [6, 11–15]. This single stranded DNA is degraded by Cas3 helicase-nuclease [16–19].

Cascade catalyses interference R-loops by a sequential process reliant on recognition of protospacer adjacent motif (PAM) sequences located immediately next to a protospacer on the target protospacer DNA [20–23]. CRISPR arrays lack a PAM sequence, helping to prevent targeting of self-DNA by Cascade [24–26]. In *E. coli* K-12 PAM 5′-CTT-3′ is most prevalent (80 %) [22, 26, 27], and of 64 possible PAMs, five PAMs are tolerated by Cascade to promote interference [11, 22, 25]. The Cse1 (CasA) subunit of Cascade is important for PAM recognition, and for positioning Cas3 to degrade invader DNA [13, 16, 19]. After PAM recognition, stable R-loop formation requires complementarity between the crRNA-DNA in a “seed” region (8-10 nucleotides from the 5′ end of the crRNA spacer sequence). Upon reaching the end of the protospacer the R-loop becomes locked [28] and by pushing Cse2 dimer it induces repositioning of Cse1 and Cse2 proteins, and conformational change of the whole Cascade complex [29]. After locking of the R-loop, additional PAM verification guides Cas3 binding near the PAM [13, 19, 29] and degradation of the DNA [11, 15, 29].

“Escape” mutations in phage DNA can reduce the stability of *E. coli* Cascade R-loops when they arise in a protospacer seed or a PAM, and correspond to reduced resistance to plaque formation [11, 21–23, 25, 30, 31]. Other mutations in phage DNA protospacers are tolerated for interference (e.g. positions 6, 12, 18, 24, 30) [22]. This is because five Cas7 proteins fold over every sixth nucleotide of the crRNA which are flipped outward and do not participate in DNA recognition [8–10]. Recent results showed that the crRNA spacer sequence also has significant role in helping interference machinery to recognize protospacer with single point mutations within the seed sequence or PAM [32]. Escape mutations that block interference promote rapid acquisition of new spacers from the same target DNA, a process called ‘primed’ adaptation that suggests cross-talk between Cas1-Cas2 DNA capture and alternative binding mode of Cascade that promotes priming [22, 27, 31]. Primed adaptation is a very robust process that tolerates up to 13 mutations either in PAM or protospacer region [22]. Spacers newly acquired during primed adaptation therefore provide elevated protection against invader whose escape mutations were evading robust interference by Cascade [27, 32].

In *E. coli* H-NS (nucleoid-structuring protein) represses transcription of CRISPR and the genes encoding Cascade, Cas1 and Cas2. *E. coli* LeuO de-represses transcription of genes encoding Cascade, by blocking cooperative spreading of H-NS along the promoter of the initially transcribed Cascade gene, *cse1* [33, 34]. In addition to H-NS and LeuO, CRP (cAMP receptor protein) is a transcriptional repressor of *cas* genes in *E. coli* [35, 36]. The CRP binding site in this case overlaps with the LeuO binding site, leading to the proposal that CRP and LeuO compete for binding to the *cse1* promoter depending on the cellular availability of cAMP [35]. In contrast to the apparent complexity of transcriptional regulation of Cascade and CRISPR, factors that control transcription of *E. coli cas3* have not been identified.

Experimental analyses of CRISPR-Cas in *E. coli* can overcome influences of H-NS, LeuO and CRP repression by engineering inducible expression of CRISPR, Cascade and Cas3 from plasmids or their chromosomal loci [27, 37]. Deletion of H-NS (Δ*hns*), or ectopic over-expression of LeuO in cells with engineered anti-λ spacer (λT3) into CRISPR, promote CRISPR interference observed as enhanced resistance to phage λ*vir* [34, 38]. In these studies the protospacer targeted by spacer λT3 crRNA had a non-consensus PAM 5′-CCA-3′ and although resistance to phage λ*vir* from these strains was effective at 30 °C, we noticed that at 37 °C cells became sensitive to plaque formation. We investigated this further in Δ*hns* cells that had acquired a new spacer (λc) targeting protospacer with the consensus PAM 5′-CTT-3′. We report that the effect of temperature on CRISPR immunity in these *E. coli* cells was correlated to expression of Cas3 and its chaperone HtpG (high-temperature protein G). Inducible ectopic expression of Cas3 in the presence, but not absence, of chromosomal *htpG* rescued resistance to phage at 37 °C. Further research will be required to uncover how temperature causes this effect on activity of the *E. coli* CRISPR-Cas system.

## Results

### Temperature-dependent resistance of Δ*hns E. coli* cells to phage λ*vir* is not caused by PAM sequence variation

Genetic analysis of *E. coli* CRISPR-Cas in previous studies established that H-NS represses transcription of the operon encoding Cascade, Cas1-Cas2 (*casABCDE12*) and CRISPR locus 2.1 [33–35, 38]. Deleting H-NS (Δ*hns*) from cells de-repressed transcription, and efficient resistance to λ*vir* infection at 30 °C was reported when CRISPR of Δ*hns* cells was engineered to contain an anti-λ phage spacer sequence (λT3) [34, 38]. The importance of the λT3 spacer was highlighted by sensitivity of Δ*hns* cells to λ*vir* plaque formation compared to Δ*hns* + λT3 cells [38]. When repeating these experiments we also observed about 100000 fold elevated resistance of Δ*hns* + λT3 cells to λ*vir* infection at 30 °C, compared to Δ*hns* cells without λT3 spacer (Table 1). However at 37 °C, in otherwise identical assays, Δ*hns* + λT3 cells became sensitive to phage (Table 1). Cells with intact H-NS (*hns*^+^), with or without λT3, were sensitive to λ*vir* infection at both temperatures (Table 1). There was no difference in sensitivity at 37 °C between Δ*hns* cells + or - λT3 spacer. If λT3 spacer was absent from the CRISPR locus 2.1, Δ*hns* cells showed threefold increase in resistance at 30 °C in comparison to 37 °C (Table 1). Therefore using 30 °C temperature of incubation in infectivity assays is an important factor for promoting resistance of Δ*hns* + λT3 cells to phage λ*vir*.

**Table 1:** *E. coli* cells lacking H-NS show temperature-dependent resistance to phage

strain	genotype	Plaque forming units (PFUs)	
		30 °C	37 °C
BW25113	*hns* ^*+*^	3.80 × 10^10^ ± 7 × 10^9^	4.56 × 10^10^ ± 9 × 10^9^
BW39121	Δ*hns*	1.20 × 10^10^ ± 1.8 × 10^9^	4.23 × 10^10^ ± 6.6 × 10^10^
BW39651	*hns* ^+^ + λT3	3.66 × 10^10^ ± 7.57 × 10^9^	4.03 × 10^10^ ± 1.2 × 10^10^
BW39671	Δ*hns* + λT3	~4 × 10^5^	4.16 × 10^10^ ± 5.5 × 10^9^

λ*vir*

Cell lawns were infected with phage dilutions (from 10^−2^ to 10^−8^) and incubated at either 30 °C or 37 °C. Cells lacking H-NS (Δ*hns*) or containing H-NS (*hns* ^+^) had fully operational CRISPR-Cas systems that were engineered with an anti-λ spacer (λT3) as indicated. The average of at least three independent experiments are shown

The λT3 spacer sequence engineered into CRISPR used here and in [37, 38] has a nucleotide sequence match with the template strand of λ phage gene lysis R, but the PAM sequence (5′-CCA-3′, Additional file 1: Figure S1A) deviates from consensus 5′-CTT-3′ *E. coli* PAM [20]. Single nucleotide variations in PAMs may disrupt interference in Type I-E, I-F and Type II CRISPR systems by preventing R-loop priming and degradation of invading DNA [21, 22, 25, 29, 30, 39, 40]. Five PAMs, CAT, CTT, CCT, CTC and CTA found previously [11, 22, 25], are utilized by Cascade for robust interference, and ten non-consensus PAMs give a partial resistance phenotype [25]. Therefore, PAM 5′-CCA-3′ belongs to the latter group, giving partial resistance to λ*vir* as expected from previous data [25]. However, recent findings showed that spacer sequence dictates whether mutant PAM sequences will be tolerated for interference or not [32]. We investigated if the observed major difference in phage resistance of Δ*hns* cells at 30 °C and 37 °C was related to PAM sequence by introducing spacer targeting protospacer with the consensus PAM 5′-CTT-3′ into CRISPR. To do this we provoked Δ*hns +* λT3 *E. coli* to acquire a new spacer. One such *E. coli* derivative containing spacer (λ*c*) targeting phage λ*vir* gene *cII* with a 5′-CTT-3′ PAM was selected. The procedure is detailed in the methods and supplementary material. The constructed strain (IIB1039; Table 2) also contains Δ*cas1* mutation as a useful controlling factor to uncouple interference from adaptation, enabling focus on interference reaction only. In phage infectivity assays, *hns*^+^ Δ*cas1* + λ*c* + λT3 cells were sensitive to λ*vir* phage at 30 °C and 37 °C, as expected because H-NS represses *cas* genes (Table 3). Δ*hns* Δ*cas1* + λ*c* + λT3 cells showed ~10^8^ fold increase in resistance at 30 °C compared to *hns*^+^ Δ*cas1* + λ*c* + λT3, and ~10^3^ compared to Δ*hns* Δ*cas1* + λT3 cells, but remained sensitive to λ*vir* infection at 37 °C (Table 3). This showed that Δ*cas1* mutation did not affect interference as expected and confirmed the importance of a consensus PAM for phage resistance in infectivity assays at 30 °C, explained in previous studies by the effect of variable PAMs on efficacy of interference reactions [25, 29]. However, added spacer targeting the consensus PAM 5′-CTT-3′ was not able to repeal the temperature dependent resistance of Δ*hns* cells to λ*vir* in these assays, which we concluded must be caused by other factor (s).

**Table 2:** List of strains used in this study

Bacterial strain	Relevant genotype	Source or reference
EB304	MG1655, Δ*cas3*::*apra*	[44]
BSN22	W3110, Δ*hns*::*cat*	[46]
BW25113	F^−^ *rrnB* Δ*lacZ4748* (::*rrnB-3*) *hsdR514* Δ (*araBAD*) *567* Δ (*rhaBAD*) *568 rph-1* λ^−^	[47]
	Bacterial strains related to BW25113	
BW39121	+ Δ*hns*::*kan*	[38]
JW0462	+ Δ*htpG*::*kan*	[41]
BW40114	+F’ (*proAB* ^+^ l*acI* ^*q*^ZΔM15::Tn*10*) *lac*UV5-*cas3 cat*::*araB*p8-*casA*	[27]
BW39651	+ λT3 spacer	[38]
BW39671	+ λT3 Δ*hns*::*kan*	[38]
BW39183	+ Δ*cas1*::*kan*	[38]
IIB848	+ λT3 Δ*cas3*::*apra*	recombineering using pKD46
IIB870	+ λT3 Δ*cas3*::*apra*	P1. IIB848 × BW39651
IIB965	+ λT3 Δ*cas1*::*kan*	P1. BW39183 × BW39651
IIB966	+ λT3 Δ*cas1*::*kan* Δ*hns*::*cat*	P1. BSN22 × IIB965
IIB969	+ λT3 *lac*UV5-*cas3 cat*::*araB*p8-*casA*	P1. BW40114 × BW39651
IIB969e	+ λc + λT3 *lac*UV5-*cas3 cat*::*araB*p8-*casA*	Selection of λ^r^ colony with phage acquired spacer
IIB1039	+ λc + λT3 Δ*cas1*::*kan*	P1. BW39183 × IIB969e (selection Km^r^ Chl^s^ and PCR of the CRISPR-1 region)
IIB1040	+ λc + λT3 Δ*cas1*::*kan* Δ*hns*::*cat*	P1. BSN22 × IIB1039
IIB1043	+λc + λT3 Δ*cas1*::*kan* ^*S*^	Removal of kan cassette by pCP20 plasmid
IIB1063	+λc + λT3 *lac*UV5-*cas3 cat*::*araB*p8-*casA* Δ*htpG*::*kan*	
IIB1065	+λc + λT3 Δ*cas1*::*kan* ^*S*^Δ*htpG*::*kan*	P1. JW0462 × IIB1043
IIB1066	+λc + λT3 Δ*cas1*::*kan* ^*S*^Δ*htpG*::*kan* Δ*hns*::*cat*	P1. BSN22 × IIB1065

**Table 3:** Temperature-dependent resistance of Δ*hns* cells to phage λ*vir* in the presence of phage acquired spacer

strain	genotype	Plaque forming units (PFUs)	
		30 °C	37 °C
IIB965	*hns* ^*+*^ Δ*cas1* + λT3	4.63 × 10^10^ ± 1.6 × 10^10^	5.8 × 10^10^ ± 1.14 × 10^10^
IIB966	Δ*hns* Δ*cas1* + λT3	~5 × 10^5^	3.35 × 10^10^ ± 9 × 10^9^
IIB1039	*hns* ^+^ Δ*cas1* + λc + λT3	3.37 × 10^10^ ± 2.5 × 10^9^	2.97 × 10^10^ ± 9.7 × 10^9^
IIB1040	Δ*hns* Δ*cas1* + λc + λT3	~ 3 × 10^2^	2 × 10^10^ ± 6 × 10^9^

Cell lawns of strains *hns* ^*+*^ (Δ*cas1*) + λc + λT3 and Δ*hns* (Δ*cas1*) + λc + λT3 were infected with phage dilutions (from 10^0^ to 10^−8^) and incubated at either 30 °C or 37 °C. The average of at least three independent experiments are shown

### Transcription and stability of *cas3* in Δ*hns* cells is limiting for resistance to phage

We tested if the effect of temperature on phage resistance was influenced by variations in expression of *E. coli* CRISPR-Cas. Robust resistance of Δ*hns* Δ*cas1* + λc + λT3 cells to λ*vir* (Table 3) suggested all components of CRISPR-Cas were expressed in sufficient amounts at 30 °C. A previous analysis identified increased transcription of genes encoding Cascade and crRNA in Δ*hns* cells grown to mid-log phase, but no such increase in *cas3* transcripts [34]. We explored if levels of Cas3 RNA or protein in cells might correlate to phage resistance under different temperature conditions of infectivity assays.

**Fig. 1:**
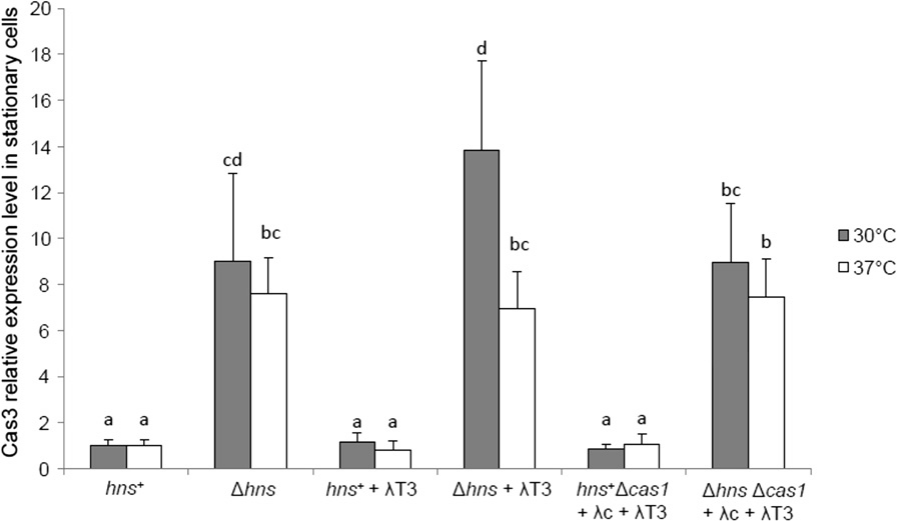
Cas3 is transcribed in cells lacking H-NS at both temperatures of incubation in stationary phase of growth. qPCR analysis extended to Δ*hns* Δ*cas1* and *hns* ^*+*^ Δ*cas1* cells containing λT3 and/or λc anti-lambda spacer. Relative expression levels of *cas3* transcripts are measured in cells grown to stationary phase at indicated temperatures of incubation with *groES* as reference gene. Error bars represent normalized error of respective duplicates. Histogram bars marked with different letters (a for *hns* ^+^ samples, and b, bc, cd and d for Δ*hns* samples) indicate significant difference between expression values, while samples that share a letter in the notation (e.g. b, bc, cd or d and cd) do not have statistically different expression values. Cas3 expression levels were compared across all samples, and two expression values were considered significantly different as evaluated by One-way ANOVA Duncan Multiple Range post-hoc test (*p <* 0.05)

**Fig. 2:**
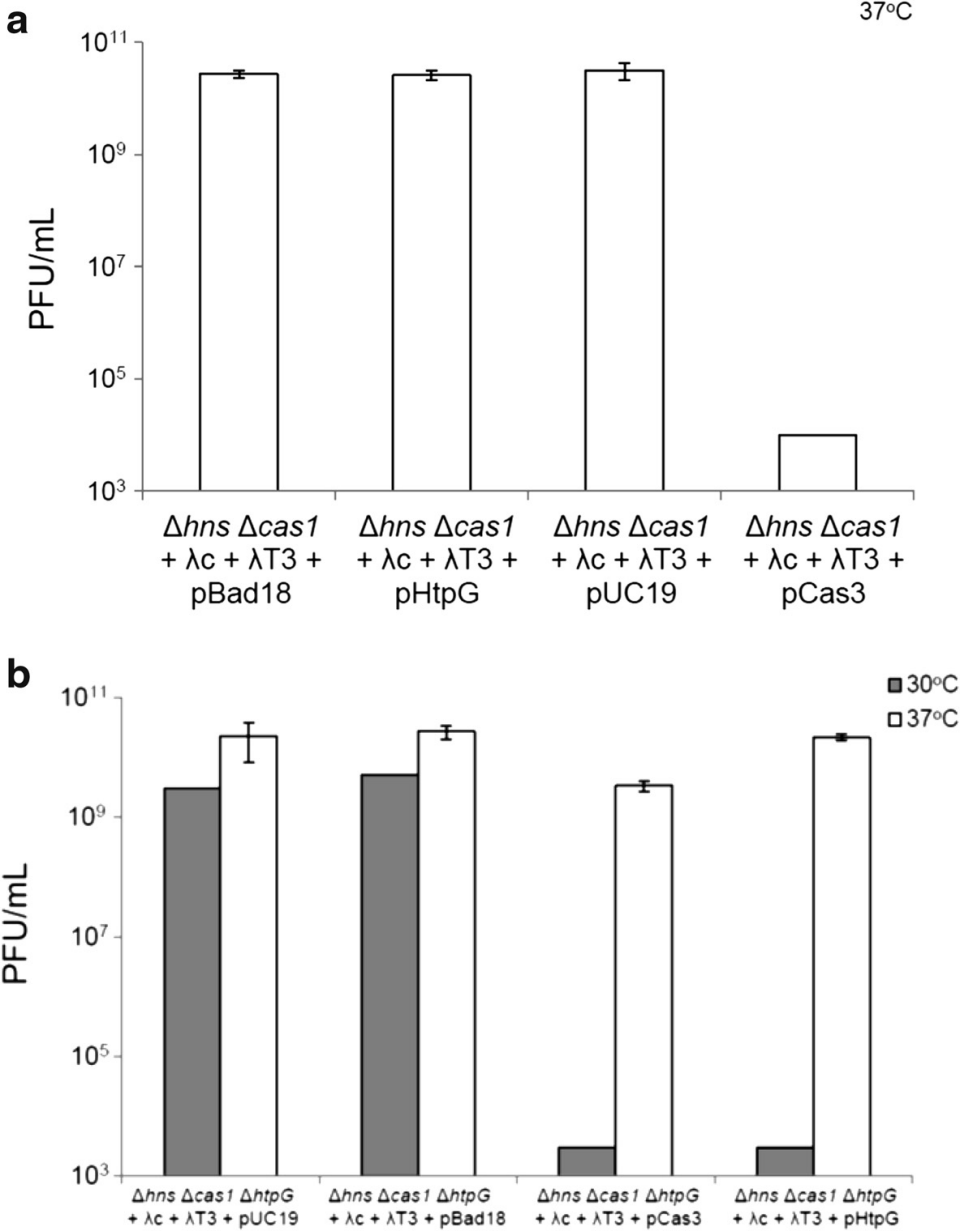
HtpG is required for resistance to λ*vir* at 30 °C and 37 °C to prevent Cas3 instability. **a** *E. coli* cell lawns of strain Δ*hns* Δ*cas1* + λc + λT3 (IIB1040) transformed with pCas3 and pHtpG expressing plasmids and empty vector controls were infected with phage dilutions (from 10^0^ to 10^−8^) and incubated at 37 °C. Bars represent average and SD of the number of plaque forming units (PFUs) per ml from three independent experiments. **b** *E. coli* cell lawns of strains Δ*hns* Δ*cas1* + λc + λT3 (IIB1040) and Δ*htpG* Δ*hns* Δ*cas1* + λc + λT3 (IIB1066) and IIB1066 transformed with pCas3 and pHtpG expressing plasmids, were infected with phage dilutions (from 10^0^ to 10^−8^) and incubated at either 30 °C or 37 °C. Bars represent average and SD of the number of plaque forming units (PFUs) per ml from three independent experiments

By using quantitative PCR (qPCR) we compared *cas3* transcript levels between *hns*^+^ and Δ*hns* cells in both mid-log and stationary phases of growth. Relative abundance of *cas3* transcripts was around eight-fold higher in Δ*hns* cells compared to *hns*^+^ cells when grown to stationary phase, regardless of the temperature being 30 °C or 37 °C (8.99 ± 3.83 and 7.59 ± 1.59), but remained similar at mid-log phase (0.9 ± 0.56 and 2.44 ± 1.21 for 30 °C and 37 °C, respectively). This suggested that the growth phase was important for the observed difference in the level of *cas3* transcripts, not the temperature of incubation. As shown in Fig. 1, only Δ*hns* + λT3 cells showed significant difference in *cas3* transcripts depending on the temperature of incubation (marked with different letters d and bc which indicate significant difference in expression values between these two samples (*p <* 0.05)). Increased *cas3* transcription at stationary phase at 30 °C or 37 °C was not observed if H-NS was present, and presence of λc + λT3 spacers had no effect on *cas3* transcript levels (Fig. 1). These results suggest that the sensitivity of Δ*hns* cells to λ*vir* plaques at 37 °C is unlikely to be due to lack of *cas3* transcription. We therefore tested if Cas3 protein levels may influence phage sensitivity at 37 °C. The chaperone HtpG was also considered here, because HtpG increases steady-state Cas3 protein levels in *E. coli*, which correlated to stimulation of interference reactions, carried out at 32 °C [41]. We reasoned that sensitivity of Δ*hns* Δ*cas1* + λ*c* + λT3 cells to λ*vir* at 37 °C caused by reduced or unstable Cas3 protein might be overcome by plasmid expression of HtpG or Cas3. However, we observed that (Fig. 2a) expression of only Cas3 from plasmid restored resistance of Δ*hns* Δ*cas1* + λ*c* + λT3 cells to λ*vir* at 37 °C comparably to at 30 °C, while cells containing empty plasmid vectors (pBAD or pUC19) or HtpG expressing plasmid remained sensitive. These results suggest that endogenous levels of functionally active Cas3 in Δ*hns* Δ*cas1* + λ*c* + λT3 cells are too low to be relieved by elevated levels of HtpG at 37 °C. Given the importance of HtpG for phage resistance it was expected that elimination of HtpG from Δ*hns* Δ*cas1* + λ*c* + λT3 cells (Δ*htpG* Δ*hns* Δ*cas1* + λ*c* + λT3) would cause sensitivity to phage at both 30 °C and 37 °C (Fig. 2b), compared to phage resistance observed in Fig. 2a. Indeed, plaques were observed at 30 °C in Δ*htpG* Δ*hns* Δ*cas1* + λ*c* + λT3 cells with or without empty plasmid controls. However, individual plaques were not visible, so the number of PFU is estimation (Fig. 2b). Plasmid expression of Cas3 (pCas3) in Δ*htpG* Δ*hns* Δ*cas1* + λ*c* + λT3 cells was sufficient for phage resistance at 30 °C (Fig. 2b) indicating that elevated amounts of Cas3 is efficient in phage defence independently of HtpG at 30 °C as shown before [41]. In contrast, elevated levels of Cas3 (pCas3) in Δ*htpG* Δ*hns* Δ*cas1* + λ*c* + λT3 cells did not rescue phage resistance at 37 °C confirming the importance of HtpG in maintaining functional levels of Cas3 in phage defence. As expected, although plasmid expression of HtpG (pHtpG) in Δ*htpG* Δ*hns* + λ*c* + λT3 cells rescued phage resistance at 30 °C, it did not at 37 °C (Fig. 2b). Given the known interplay of HtpG and Cas3 in promoting CRISPR interference in *E. coli*, these results suggest that levels of Cas3 protein are the limiting factor for resistance to λ*vir* phage infection in Δ*hns* cells at 37 °C. Overall, these results show that endogenous levels of Cas3 are expressed in low but sufficient amounts for the CRISPR-Cas mediated immunity in cells lacking H-NS grown to stationary phase at 30 °C, but that the levels of functionally active Cas3 becomes limiting at 37 °C and require increased levels of Cas3.

## Discussion

By manipulating the expression of H-NS and CRISPR-Cas in *E. coli* cells we identified that stability or activity of Cas3, with HtpG present, is a limiting factor for resistance to phage λ*vir* at 37 °C. Our genetic analyses of CRISPR interference at 30 °C agreed with previous studies, by observing robust phage resistance when cells were lacking H-NS repressor (Δ*hns*), and when an anti-λ spacer could target protospacer DNA next to a consensus PAM, 5′-CTT-3′. However, the same assays at 37 °C resulted in a dramatic loss of phage resistance that had not been observed previously. Phage resistance could be restored at 37 °C, to levels comparable with resistance at 30 °C, by inducible expression of *cas3* from plasmids.

Previous analyses of *cas3* transcription sampled only during mid-log growth and showed no difference between Δ*hns* cells compared to *hns*^*+*^ cells, unlike Cascade genes and crRNA that were much increased in Δ*hns* cells [34]. We measured *cas3* transcripts in mid-log and stationary phase, observing that Δ*hns* cells contained eight-fold more *cas3* transcript compared to *hns*^*+*^ cells at 30 °C and 37 °C*.* This is significant because infectivity assays for measuring resistance of *E. coli* to λ*vir* use cells in stationary phase. We conclude from this analysis that H-NS regulates expression of the *cas3* gene, as well as Cascade and crRNA, but possibly does so under more specific growth conditions.

Recently it was highlighted that Cas3 requires HtpG chaperone for CRISPR interference assays [41]: Overexpression of HtpG or Cas3 from plasmids in *htpG* deficient cells (Δ*htpG* Δ*hns*) rescued transformation-efficiency at 32 °C. We observed similar interplay of HtpG and Cas3 at 30 °C because plasmid overexpression of either HtpG or Cas3 (pHtpG/pCas3) could restore phage resistance to Δ*htpG* Δ*hns* Δ*cas1* + λ*c* + λT3 (IIB1066) cells that were otherwise sensitive to phage infection (Fig. 2b). Interestingly, the pCas3 alone did not restore phage resistance to Δ*htpG* Δ*hns* Δ*cas1* + λ*c* + λT3 cells at 37 °C (Fig. 2b) but did to *htpG*^+^ Δ*hns* Δ*cas1* + λ*c* + λT3 (IIB1040) cells at 37 °C (Fig. 2a), while pHtpG was unable to sustain phage resistance to any Δ*hns* cells at 37 °C. Thus, HtpG is important for CRISPR-system activity in Δ*hns* cells at 37 °C, but its overexpression from plasmid alone cannot overcome limiting amounts of functionally active Cas3 at 37 °C. Further research will be required to better understand the reasons and mechanisms of Cas3 instability in Δ*hns* cells at 37 °C.

In addition to four σ^70^ (“house-keeping” sigma factor)-promoters in CRISPR-Cas area, two potential σ^32^ (heat-shock sigma factor)-dependent promoters have been mapped within coding regions of *cas7* and *cas1* [42, 43], suggesting another possible link between CRISPR-Cas immunity and heat-shock response. In summary, expression and activity of the CRISPR-Cas system in *E. coli* seem to be linked to global stress responses, such as H-NS global repressor, heat stress and CRP-cAMP. Perhaps, CRISPR-Cas immunity is designed to become activated during certain phase of growth, at specific environmental habitats and temperature, and instability of the Cas3 may be the mechanism for inactivation of the CRISPR-Cas defence either at inappropriate temperature of incubation or when degradation of foreign DNA is completed.

## Conclusions

We observed that the ability of an *E. coli* CRISPR-Cas system to resist lysis by phage λ was strongly influenced by temperature. Genetic analysis of this effect indicated that sensitivity to phage at 37 °C was caused by limiting amounts of Cas3, rather than effects of PAM sequence variations on Cascade interference reactions. We show that transcription of *cas3* is controlled by H-NS: elimination of H-NS from cells correlated to eight-fold increased levels of *cas3* transcript, specifically in stationary phase growth. At 37 °C, increased expression of *cas3* is required for resistance to λ infection. This suggests that endogenous expression and activity of Cas3 is responsive to signals associated with growth phase and temperature in *E. coli*.

## Methods

### Strains and plasmids

The *E. coli* K-12 strains used in this study are described in the Table 2.

Plasmids used were: pEB526 expressing Cas3 [44], and HtpG expressing plasmid was from pBAD18 plasmid [41].

### Media and general methods

LB broth and agar media (10 g L^−1^ bacto-tryptone, 5 g L^−1^ yeast extract, 10 g L^−1^NaCl), supplemented with 15 g of agar for solid media. When required appropriate antibiotics were added to LB plates at final concentrations: ampicillin at 100 μg/ml, kanamycin at 40 μg/ml, apramycin 30 μg/ml and chloramphenicol at 15 μg/ml. Mutant bacterial strains were made by P1*vir* transduction and selected for the appropriate antibiotic resistance [45]. When important (for generating IIB969 and IIB1039), the genotype (presence of λT3 and λc spacers) of many transductants were screened by colony PCR using the same primers (CRISPR I-R: 5′-GAGATGCAGGCCATCGGA-3′ and spacer 4: 5′-GCGACCGCTCAGAAATTCCAGACCCGATCCAAA-3′) as for spacer acquisition and PCR products were sequenced for confirmation.

### Phage sensitivity assay by plaque formation

Cells were grown to saturation overnight in LB medium supplemented with 0.02 % maltose. LB plates were overlaid with 3.5 ml 0.6 % LB top agar containing 0.2 ml of cells. After solidification, 10 μl aliquots of serially diluted phages in 10 mM MgSO_4_ were spotted on the surface of the plate and allowed to soak. Plates were incubated overnight at 30 °C or 37 °C. When required, 1 mM IPTG (isopropyl-β-D-thiogalactoside) and 0.2 % L-arabinose were added in plates and top agar. The sensitivity of the cells to infection was represented as the plaque-forming units (PFUs) by counting plaques from several dilutions, and calculating their number per mL.

### Spacer acquisition experiments to generate a consensus PAM for interference

Spacer acquisition was performed according to [27]. Strain IIB969 with *cas* genes fused to inducible promoters and containing the anti-lambda λT3 spacer in CRISPR locus 2.1 (Table 2) was grown at 37 °C at 200 rpm to log phase in LB medium containing 1 mM IPTG and 0.2 % L-arabinose until OD_600_ was 0.4-0.5, and mixed with λ*vir* lysate at appropriate MOI = 1. Cell-phage mixture was incubated for 15 minutes without agitation at 37 °C. The mixture was then diluted 10 fold with fresh LB medium containing the same inducers and incubated at 37 °C for at least two hours, in most cases overnight. Aliquots were spread on LB plates with IPTG and arabinose and incubated overnight at 37 °C. CRISPR expansion was monitored by PCR using appropriate pairs of primers specific for CRISPR locus 2.1 mentioned above.

Several PCR products of fragments of CRISPR locus 2.1 (Additional file 1: Figure S1B) were sent for sequencing in Macrogene service. One lambda resistant derivative was kept for further research (IIB969e, Table 2). It contained phage-acquired anti-λ spacer targeting λ*cII* gene (called λ*c)* with consensus PAM 5′-CTT-3′ (Additional file 1: Figure S1C). Δ*hns* + λ*c* + λT3 strain was made by P1 transduction using donor strain Δ*cas1*:: Km^r^ (BW39183) and selecting for *hns*^+^ recombinant strain with two extra spacers in the CRISPR locus 2.1 (strain IIB1039; Table 2) and later introducing Δ*hns* mutation (strain IIB1040; Table 2). The Δ*cas1* mutation was chosen to create Δ*hns* + λ*c* + λT3 cells because *cas1* gene is not required for interference [37].

### RNA extraction and qPCR

Total RNA was extracted from mid log (OD_600_ = 0.4-0.5) and overnight cultures incubated at 30 °C or 37 °C. 1.5 ml of each culture was used and the cell pellet was resuspended in cold 10 mM EDTA and 50 mM sodium citrate and Trizol LS (Invitrogen) was used to extract total RNA following the instructions from the manufacturer. The same amounts of RNA (1 μg) was first treated by DNase I, diluted 10 fold and 2 μl of each sample (in duplicate) was used as a template for one step amplification reaction using One Step SYBR Prime Script RT-PCR Kit II (Takara Bio. Inc.). The PCR reactions were performed on a 7500 Fast Real Time PCR System (Applied Biosystems) and analysed using 7500 Software v.2.0.6. (Applied Biosystems). As an internal control the *groES* gene was used. Fold change of the *cas3* gene transcription was calculated using relative quantification with *groES* as endogenous control and *cas3* gene transcript from *E. coli* BW25113 (wild type) abundance as calibrator. All PCR reactions were performed in triplicate. Control PCRs without template were performed to monitor general contamination levels. Results of qPCR (ΔCt values) were analyzed by one-way analysis of variance (ANOVA) using STATISTICA 12.0 (StatSoft Inc, USA) software package. Duncan Multiple Range Test was used for post-hoc analysis. Differences between two sample means were considered statistically significant at *p <* 0.05.

Primers used were:

- Cas 3-F: 5′-ATCGCGTCAATGTACCCTTC-3′
- Cas3-R: 5′-TCCAGCCAAAGTAACCCATC-3′
- groES-F: 5′-CTG GAT CGT CAA GCG TAA AG-3′
- groES-R: 5′-CAA GGA TAC GGC CAT TGC-3′

## Additional file

Additional file 1: Figure S1. Properties of PAM in λT3 spacer and phage acquired spacer (A). Sequence complementarity between λT3 spacer and proto-spacer in lambda with the PAM 5′ CCA 3′ highlighted in red type. (B) Detection of new spacers acquired in CRISPR locus 2.1. The agarose gel shows products of PCR amplified CRISPR 2.1 from *E. coli* cells IIB969 (lane C “control”), and two phage resistant derivatives after phage challenge of IIB969 (lanes a and b). The PCR fragment size is 723 bp before and 784 bp after spacer acquisition (C). λc spacer sequence from the strain IIB969e is presented paired with proto-spacer in lambda (targeting gene *cII*) with the PAM 5′-CTT-3′ highlighted in red type. (JPG 311 kb)

## Abbreviations

cAMP: cyclic AMP
Cas: CRISPR-associated
Cascade: CRISPR-associated complex for antiviral defence
CRISPR: clustered regularly interspaced short palindromic repeats
CRP: cAMP receptor protein
crRNA: CRISPR-RNA
E. coli: Escherichia coli
H-NS: nucleoid-structuring protein
HtpG: high-temperature protein G
MOI: multiplicity of infection
OD: optical density
PAM: protospacer adjacent motif
PFU: plaque forming units
λ: lambda
