# Supplementary figures and images for: Cas3 is a limiting factor for CRISPR-Cas immunity in Escherichia coli cells lacking H-NS
Source: BMC Microbiol. 2016 Mar 8;16:28. doi: 10.1186/s12866-016-0643-5 (PMC4782391; doi:10.1186/s12866-016-0643-5)

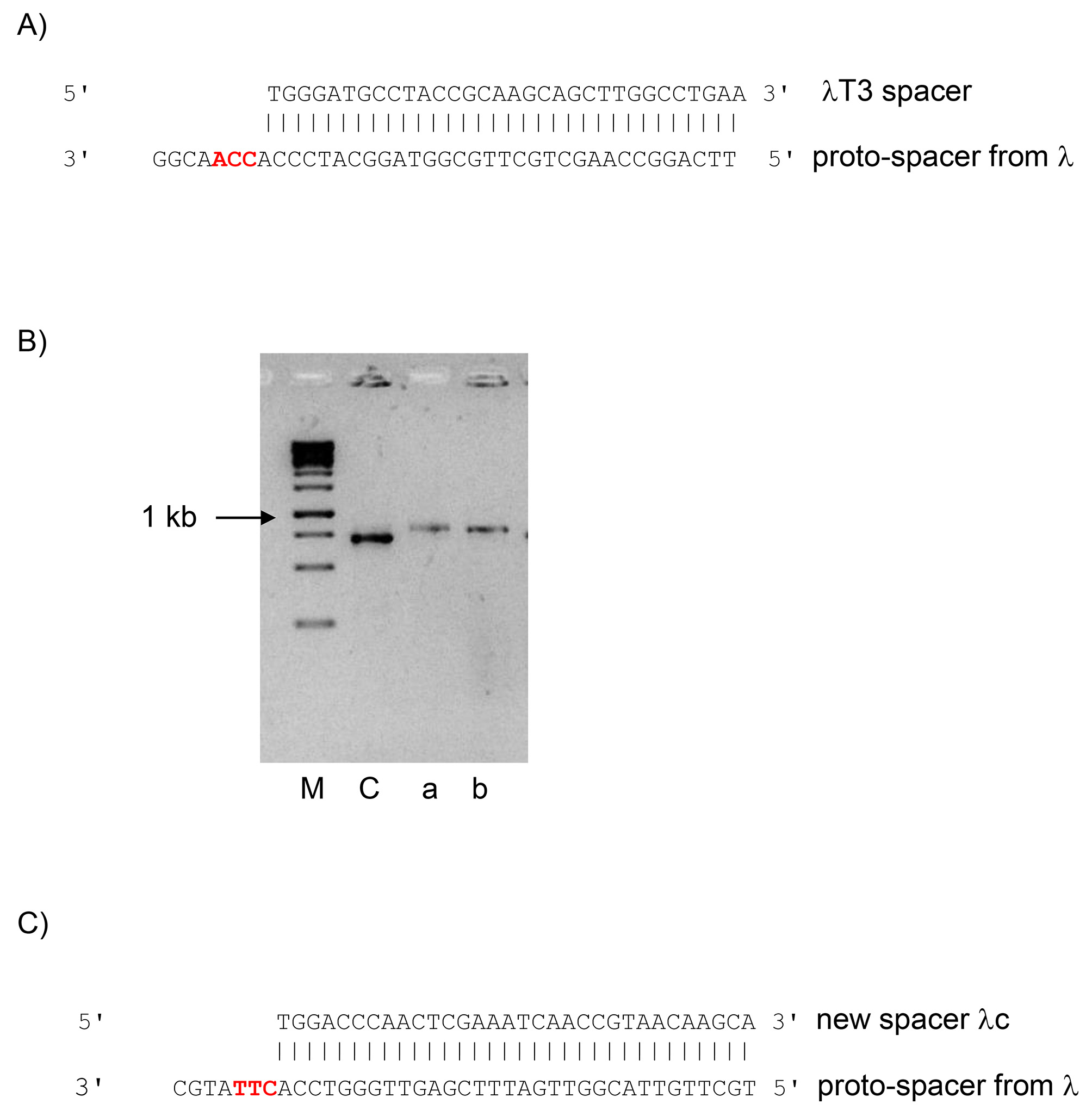

Supplement: Additional file 1: Figure S1. — Properties of PAM in λT3 spacer and phage acquired spacer (A). Sequence complementarity between λT3 spacer and proto-spacer in lambda with the PAM 5′ CCA 3′ highlighted in red type. (B) Detection of new spacers acquired in CRISPR locus 2.1. The agarose gel shows products of PCR amplified CRISPR 2.1 from E. coli cells IIB969 (lane C “control”), and two phage resistant derivatives after phage challenge of IIB969 (lanes a and b). The PCR fragment size is 723 bp before and 784 bp after spacer acquisition (C). λc spacer sequence from the strain IIB969e is presented paired with proto-spacer in lambda (targeting gene cII) with the PAM 5′-CTT-3′ highlighted in red type. (JPG 311 kb) [file 12866_2016_643_MOESM1_ESM.jpg]
